# Peer Effects on Safety and Productivity in a Simulated Workplace Through Usability and Ecological Validity Testing of a Nonimmersive Virtual Simulation With University Students: Cross-Sectional Qualitative Study

**DOI:** 10.2196/81061

**Published:** 2026-07-31

**Authors:** Chukwudiebube Atagbuzia, Ean H Ng, Ganapathy Natarajan, Javier Calvo-Amodio, Zlata Goleva

**Affiliations:** 1 Oregon State University Corvallis, OR United States; 2 University of Idaho Moscow, ID United States; 3 University of Wisconsin–Platteville Platteville, WI United States

**Keywords:** ecological validity, nonimmersive virtual simulation, peer effects, safety behavior, usability

## Abstract

**Background:**

Peer effects influence workplace safety and productivity as employees observe and compare themselves to coworkers. However, prior simulation research has focused mainly on productivity in immersive settings, with limited attention to safety and little evidence on whether nonimmersive environments can capture peer effects across both domains. This study developed a nonimmersive virtual simulation to model peer effects on safety and productivity behavior.

**Objective:**

This study aimed to assess the usability and ecological validity of a theoretically grounded nonimmersive virtual simulation designed to model peer effects through exposure to virtual coworker performance information.

**Methods:**

A cross-sectional qualitative design was used across 2 iterative testing phases, alpha and beta. Participants were university students aged 18-30 years (alpha: n=12; beta: n=12). Alpha phase participants had prior kitchen experience to support early usability testing, while beta phase participants reflected the broader target population. The simulation involved timed meal preparation tasks with integrated safety requirements and peer performance feedback. Gameplay observations and semistructured interviews examined instructional clarity, environmental realism, virtual peer credibility, and scoring design.

**Results:**

Alpha phase participants struggled with written instructions, perceived limited realism, and found peer comparisons ineffective because peer scores were unrealistic. After revisions, including experiential training, calibrated peer scores, clearer peer source messaging, and improved environmental cues, beta phase participants reported clearer instructions, greater realism, and more motivating peer comparisons.

**Conclusions:**

Results support that a theoretically grounded nonimmersive simulation refined through evidence-based feedback can elicit meaningful peer-related responses in safety and productivity contexts. By preserving calibrated social comparison, credible peer representation, and realistic task contingencies, the simulation offers a controlled and scalable way to examine how peer information shapes safety productivity dynamics. These findings expand methodological options for studying peer effects in settings where real-world experimentation may be impractical or unsafe.

## Introduction

### Background

Peer effects impact both productivity [1-3] and safety [4-6]. Employees tend to adjust their effort based on the performance of those around them, and observing coworkers ignore safety rules increases the likelihood of similar behavior [4-6]. Although studies have shown peer effects on productivity and safety separately, these outcomes are interdependent in practice [7-9]. Productivity and safety often compete or trade off in real work settings, making it important to understand how peer behavior may impact both domains simultaneously to inform more effective intervention strategies. Incentives have also been shown to influence behavioral change in the workplace, particularly in promoting productivity [10].

Research on peer effects has grown in behavioral economics, but studying these dynamics is methodologically challenging [11]. For safety behaviors, manipulating peer conditions can introduce ethical concerns and physical risk [12,13]. Virtual environments allow researchers to control social exposures while eliminating real-world danger [14]. Prior studies have used virtual reality (VR) to investigate behavioral outcomes, including teamwork [15], performance [16,17], and social learning [18]. Behaviors and knowledge gained within virtual simulations have also been shown to transfer effectively to real-world settings [19-23], suggesting that when simulations are ecologically valid, they can reliably predict actual behaviors. Virtual peers are especially useful for studying social effects because they allow researchers to separate the influence source from the receiver [16,17], addressing Manski’s [11] reflection problem.

However, achieving and validating ecological validity can be complex. Ecological validity refers to the extent to which results observed in experimental or simulated settings generalize to real-world contexts [24,25]. Ecological validity has been conceptualized as a multidimensional construct defined by 3 elements: the research setting, the stimuli, and the participant’s task or response [26]. The research setting dimension concerns whether the experimental environment realistically represents the natural context in which the behavior occurs, while the stimuli and response dimensions address whether the reactions and observed behaviors correspond to those found in real-world conditions. These dimensions are consistent with later operational definitions of ecological validity. Specifically, the setting dimension aligns with verisimilitude, which refers to the similarity between simulated and real-world processes and conditions, whereas the stimuli and response dimensions align with veridicality, which captures the correspondence between simulated and real-world outcomes [25,27]. In this study, ecological validity was operationalized through verisimilitude and veridicality, reflecting how realistically the simulation represented workplace conditions and how closely participants’ perceptions and behaviors aligned with real-world peer effects.

Previous studies have suggested several methods to evaluate ecological validity. One approach is transfer or criterion correlation, which tests whether performance or behavioral outcomes in simulations correspond to those observed in equivalent real-world tasks [25,27]. Field validation or deployment studies examine whether the same behavioral effects emerge when the simulation is applied in natural environments [28]. Benchmarking methods involve comparing behavioral data from simulations with archival or logged real-world datasets to assess alignment in distributions and effect sizes [27]. Participant realism checks capture subjective or behavioral indicators of realism by asking participants to describe or rate the plausibility of the simulation or by observing whether they act naturally [28]. Finally, multicontext replication evaluates whether findings generalize across settings, populations, and devices, offering further evidence of ecological robustness [25]. Ecological validity can thus be evaluated using any of these methods individually or through a combination tailored to the research goals. The choice of method often depends on the study design, available resources, and the stage of validation [24,25]. Early exploratory studies may emphasize participant realism or incremental fidelity testing, while later-stage evaluations might rely on transfer tests or field validation [25]. Together, these complementary approaches provide a comprehensive framework for assessing how well simulation findings extend to real-world contexts.

While immersive VR provides access to advanced technologies that can enhance realism and facilitate ecological validity [23], it is important to note that ecological validity is rooted in theory rather than technology. Immersive systems can reproduce complex sensory and social contexts, but they require specialized hardware and higher costs [29,30]. Nonimmersive setups, such as desktop-based simulations, can also achieve ecological validity when they are designed around sound theoretical frameworks and realistic behavioral principles. In this sense, realism derived from technology does not guarantee ecological validity; rather, it is achieved when the simulated environment, tasks, and social interactions are theoretically grounded and representative of the real-world phenomena being studied.

This study is the first in a planned series examining how peer behavior may impact safety and productivity in virtual work settings. In this study, we assess whether a nonimmersive simulation titled PrepMaster is usable and ecologically valid for studying peer effects and incentive structures in a simulated commercial kitchen.

### Objective

Peer effects may impact both safety and productivity simultaneously, but there is a lack of studies that examine how social influence, such as peer effects, operates across these domains simultaneously. This study addresses that gap by using a nonimmersive virtual simulation to model an environment that induces peer effects on both safety and productivity.

## Methods

### Overview of PrepMaster Simulation

**Figure 1:**
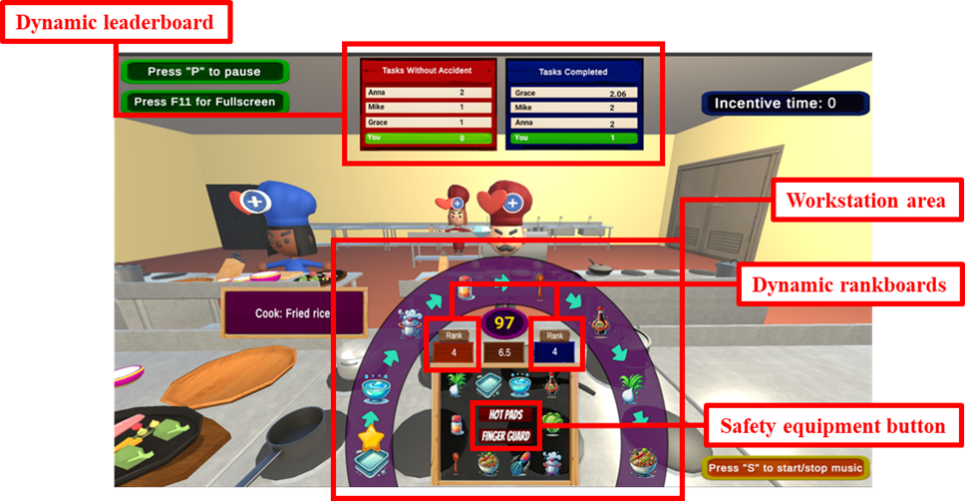
PrepMaster game interface showing the task layout, safety indicators, and peer performance display components.

PrepMaster is a first-person simulation game where participants complete a series of puzzle-based cooking tasks in a virtual kitchen environment. Throughout the game, they are given opportunities to voluntarily engage in 2 safety behaviors: wearing a knife guard before using a knife and using a hot pad before handling heated items. Each game stage requires matching visual puzzle prompts to target images in a cooking interface, simulating realistic food preparation procedures (Figure 1). The game system tracks the ratio of the number of correct safety tool usages to the number of complete tasks in a stage (Sratio).

Peer effects were introduced through information transmission in the form of a dynamic leaderboard that displays the real-time performance metrics of virtual peers, as shown in Figure 1. These metrics included safety and productivity scores updated in real time during task performance. Peer performance was manipulated as 2 independent variables: peer productivity information and peer safety information, each with 3 levels, high, low, or zero. In the high condition, virtual peers display higher average performance than the participant; in the low condition, their scores are lower; and in the zero condition, peer performance data are not shown.

A third independent variable was the productivity incentive, which had 2 levels: present or absent. When present, participants receive a 6 percent bonus to their productivity score if they complete a task faster than their own baseline speed, which is recorded during the control tasks performed in the absence of peers and peer information.

Average task completion time (Tavg) across a gameplay stage was measured as productivity. Together, these independent variables (peer productivity information, peer safety information, and productivity incentives) are designed to test their effects on the 2 outcomes: productivity and safety behavior.

### Theoretical Foundations for PrepMaster Peer Effects Design

The peer effects mechanism in PrepMaster was designed using a theory-driven model of social influence. Specifically, peer effects were implemented through information transmission and the presence of virtual peers, both intended to activate processes of competition-driven social comparison and social learning. These theoretical constructs were operationalized in the simulation through leaderboards and rank boards, which displayed participants’ performance alongside that of virtual peers, as shown in Figure 1. This design allowed participants to observe peer productivity and safety outcomes in real time, creating opportunities for comparison, motivation, and behavioral adjustment consistent with social comparison and social learning theory. The underlying idea is that individuals adjust their behavior based on how they interpret and internalize performance information from others within their environment.

**Figure 2:**
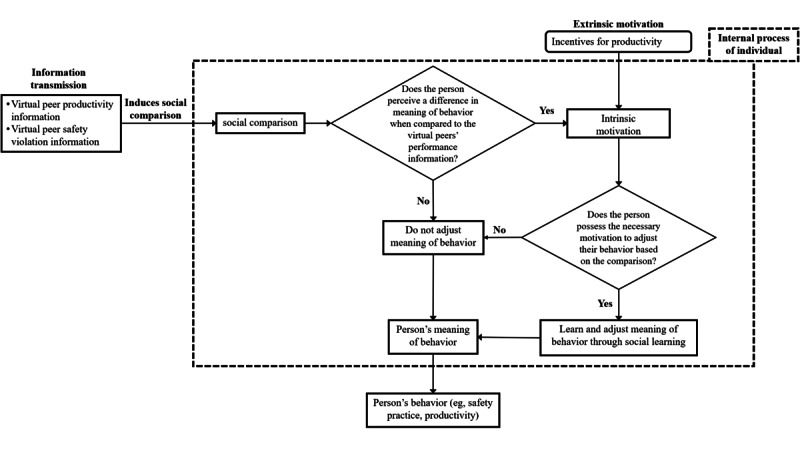
Conceptual model outlining the working process of peer effects, illustrating how exposure to peer information shapes motivation and decision-making, ultimately influencing behavioral responses.

As shown in Figure 2, the conceptual model guiding the design of peer effects in the simulation draws on psychological and social theories to explain how peer behavior might impact user actions. The process begins with exposure to peer information such as safety violations or productivity scores. According to social comparison theory, individuals assess the adequacy of their own behavior by comparing themselves to others, particularly in uncertain or novel situations [31-33]. Through social comparison, users can engage in competition if they possess the necessary motivation and self-efficacy [34-36].

After comparison, the model incorporates principles from Bandura’s [37,38] social learning theory, which states that behavioral adoption also depends on motivation and self-efficacy. Even when a user notices a peer outperforming them or behaving more safely, they must believe they can match the behavior and be motivated to do so. Without these factors, observation alone is unlikely to produce change. According to self-determination theory, extrinsic motivation such as rewards or recognition can either undermine or enhance intrinsic motivation depending on how it is perceived [39,40]. When extrinsic motivators are internalized and support an individual’s sense of autonomy and competence, they can strengthen intrinsic motivation rather than diminish it [39,40]. In VR-based training studies, increases in perceived self-efficacy and autonomous motivation have been linked to improved behavioral outcomes [41,42].

Building on these foundational theories, meaning serves as the interpretive mechanism that connects perception to action. Meaning gives reasoning to the acts performed [43], and behavior is a materialization of meaning [44]. In this context, peer comparisons not only inform self-evaluations but also shape the perceived meaning of one’s performance relative to others. This meaning determines whether the comparison is viewed as motivating, discouraging, or irrelevant, guiding how users translate peer observations into behavioral intentions.

The final step in the model is behavior change, where individuals who possess both the belief and motivation to act on peer information adopt or avoid behaviors based on what they learn. In PrepMaster, this theoretical structure informed how peer productivity and peer safety information were displayed.

### Application of Conceptual Model in Simulation Design

The conceptual model guiding this study posits that peer effects emerge through a sequence of information transmission, social comparison, and behavior change. In PrepMaster, this model was applied to peer safety by transmitting real-time data on virtual peers’ safety behavior, displayed as icons and scores representing information transmission, the first mechanism to induce peer effects, as shown in Figure 1. To ensure that participants perceived this information as relevant and competitive, peer behavior was calibrated relative to each participant’s own performance rather than to fixed high or low benchmarks. Through this adaptive information transmission, social comparison was triggered, and the incentive system activated motivation, together forming the basis for veridicality, which reflects how participants’ perceptions and reactions to peer information and the incentive system aligned with real-world peer effects and incentive dynamics.

Prior studies have relied on static peer performance definitions (eg, fast vs slow [16,17], levels of snack intake [45], and safety behavior [46]) which may be sufficient in short, single-task contexts. However, in extended simulations involving multiple tasks and potential learning effects, such static comparisons risk becoming irrelevant over time [47]. Furthermore, consistent with social comparison theory, individuals are most likely to compare themselves to others perceived as similar in ability [31,48,49]. This study addressed these concerns by defining peer productivity using a personalized 30% bias factor based on participants’ baseline scores from the control conditions, thereby inducing both upward and downward comparisons on productivity throughout gameplay.

To operationalize this feedback, virtual peers’ safety behaviors and task completion times were generated using triangular distributions, which is an approach appropriate for simulations with limited data points, using participant-specific minimum, maximum, and most likely values [50].

Tables 1 and 2 present the parameter values used to generate triangular distributions for virtual peers’ task completion times and safety behaviors. The subsequent sections detail the algorithms that governed how virtual peer performance and incentive delivery were calculated.

**Figure 3:**
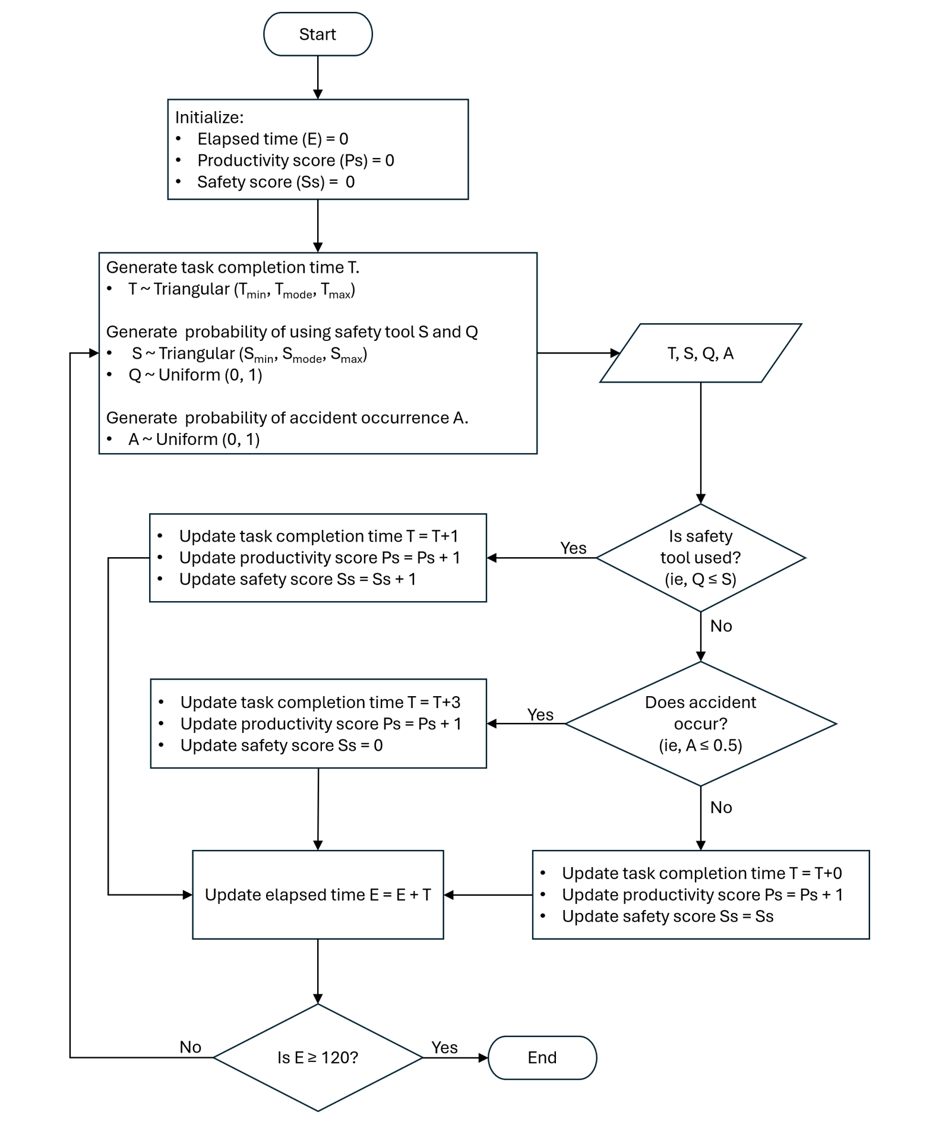
Algorithm used to generate virtual peers’ productivity and safety scores within the virtual simulation.

The algorithm to update the virtual peers’ productivity score and the probability of using safety equipment is also generated based on predetermined probabilities, and it is shown in Figure 3.

**Table 1:** Parameter definitions for generating virtual peer task completion time (T), including the high, low, and zero-productivity settings used to model different peer performance profiles.

Condition	T_min_	T_mode_	T_max_
High productivity	Tavg_c_^a^ × (1 – 0.3)^2^	Tavg_c_ × (1 – 0.3)	Tavg_c_ × (1 + 0.3)
Low productivity	Tavg_c_ × (1 – 0.3)	Tavg_c_ × (1 – 0.3)	Tavg_c_ × (1 + 0.3)^2^
Zero productivity	Tavg_c_ × (1 – 0.3)	Tavg_c_	Tavg_c_ × (1 + 0.3)

^a^Tavg_c_ is the average task completion time in the control condition.

**Table 2:** Parameter definitions for generating virtual peer safety behavior (S), including the high, low, and zero-safety settings used to model different peer performance profiles.

Condition	S_min_	S_mode_	S_max_
High safety	0.5	0.75	1.0
Low safety	0.25	0.5	0.75
Zero safety	0.2	0.625	1.0

### Research Design

This study used a cross-sectional design involving interviews and structured gameplay observation to assess the usability and ecological validity of the PrepMaster simulation. These methods were chosen because they provide detailed insight into participants’ perceptions, decision-making, and behavioral engagement within the simulated environment. Qualitative approaches are particularly valuable in early stages of evaluation, when the goal is to understand how users interpret and interact with the simulation, before proceeding to larger-scale quantitative validation [51]. Gameplay observation revealed naturalistic behaviors during task performance, while interviews captured participants’ reflections on how closely the simulation resembled real-world settings and interactions. Together, these methods offered a contextually grounded assessment of ecological validity suitable for this study’s formative phase.

An alpha test was conducted to evaluate core functionalities, while a beta test was implemented to assess the game in its final developed stage, as recommended by Lewis [52]. Two researchers conducted interviews and recorded field notes based on participant responses and observed behavior during gameplay. Both tests aimed to assess four core areas of the simulation: (1) clarity of instructions, (2) realism of simulation environment—verisimilitude, (3) peer effect design—veridicality, and (4) incentive system design. Table 3 shows a concise description and ideal state of the 4 core game areas and how they guided the qualitative analysis in the alpha and beta tests.

**Table 3:** Key game areas and their ideal states used as the evaluation criteria for assessing simulation usability and ecological validity.

Evaluated game area	Description of what was assessed	Ideal state
Clarity of instructions	How easily participants understand task goals, controls, and safety steps when first entering the simulation	Users can easily understand the goals and perform required actions [53].
Realism of simulation environment (verisimilitude)	Degree to which tasks, tools, and environment resemble real commercial-kitchen work	Participants report that actions and pacing feel authentic, and observed behaviors align with those seen in comparable real-world settings, supporting transfer of learning from simulation to the real-world [25].
Ecological validity: Peer-effect design (veridicality)	Effectiveness of leaderboard information in triggering comparison and competition	Virtual peer scores are calibrated just above or below participant’s baseline, so users perceive them as attainable yet challenging. This upward and downward comparison produces motivation to outperform or underperform peers and adopt their performance [54].
Incentive system design	Ability of the 6% productivity bonus to motivate effort without undermining safety	Users are motivated to improve their performance, consistent with goal setting and incentive theory that modest, clear rewards enhance performance [55].

### Participant Recruitment

The alpha test involved a targeted sample consisting of individuals with commercial kitchen experience and industrial engineering graduate students, selected to identify early usability issues and inform initial design revisions. The beta test was conducted with a separate group composed primarily of undergraduate students to better reflect the population intended for the main experiment. This beta test was used both to validate changes made after the alpha test and to serve as a simulated run of the main experiment to identify any remaining procedural concerns.

Participants for both tests were recruited through campus research platforms and supplemented with snowball sampling, and no incentives were offered for participation. In line with established usability research demonstrating that approximately 5 participants can uncover 80% of usability issues, with diminishing returns thereafter [56], each test targeted a sample size of 10-15 participants to ensure adequate coverage of usability and ecological validity concerns. Alpha-test participants were intentionally selected for their prior kitchen experience and familiarity with occupational workflows, whereas beta-test participants represented a broader sample of the target population.

The simulation was developed through an iterative testing process in which findings from each round informed subsequent refinements. Problems such as unclear instructions and ineffective peer modeling were identified, revised, and then reevaluated in the following round. This iterative approach is valuable in simulation development because it enables continuous improvement while ensuring that revisions are grounded in observed user behavior and participant feedback [57].

### Study Procedure

In the alpha test, participants were deliberately assigned to specific gameplay conditions to ensure that each type of peer information was tested multiple times. This included at least 2 participants each in the following conditions: (1) high peer productivity, (2) low peer productivity, (3) high peer safety, (4) low peer safety, and (5) combined peer productivity and safety information. This structured assignment allowed researchers to systematically assess how different peer information formats influenced usability and perceived realism.

For the beta test, the simulation was deployed in its final form, using an embedded randomization script designed to match the structure of the planned main experiment. Participants in this phase were randomly assigned to treatment conditions using the simulation’s inbuilt randomizer. This allowed for evaluation of usability and ecological validity under realistic gameplay conditions while preserving experimental control.

### Data Collection and Analysis

Data was collected through direct observation of gameplay and semistructured interviews conducted immediately after each test. Observational notes documented participants’ interactions with the simulation, including task strategies, safety behaviors, responses to peer information, and usability issues that emerged during gameplay. Following gameplay, participants completed a 20- to 30-minute semistructured interview focused on four domains: (1) instructional clarity, (2) environmental realism, (3) perceived credibility and impact of virtual peers, and (4) incentive and scoring design. Interviews were audio-recorded with permission and transcribed verbatim.

Analysis involved reviewing observational notes and interview transcripts to identify recurring themes within the 4 assessed domains. Transcripts were read multiple times to extract patterns in participants’ interpretations, areas of confusion, perceived realism, and reactions to peer-performance information. Themes from the alpha test informed design revisions, and the same thematic structure was applied in the beta test to assess whether these revisions improved usability and ecological validity. This approach provided a systematic means of evaluating user experience, determining the effectiveness of design changes, and assessing whether the simulation supported the intended behavioral and perceptual responses related to safety, productivity, and peer effects.

### Ethical Considerations

This study was conducted as a program evaluation to assess the functionality, usability, and technical performance of the virtual simulation rather than as a systematic investigation designed, at the outset, to develop or contribute to generalizable knowledge. For this reason, institutional review board (IRB) ethics approval was not sought before the evaluation began. This determination was based on 45 CFR 46.102 [58], which defines the regulatory scope of human subject research, and on the author’s university Human Research Protection Program guidance, which identifies program evaluation as an activity considered for IRB review based on its design and intent.

After the evaluation was completed, the author’s university Human Research Protection Program confirmed that the study did not constitute human subjects research and therefore did not require IRB ethics approval. The evaluation was limited to assessing the simulation environment and procedures, and no identifying information, identifiable images, or private identifiable participant data were collected or included in the manuscript or supplementary materials. Participants were informed of the purpose and procedures of the evaluation, their participation was voluntary, and they could withdraw at any time without penalty. Participants did not receive compensation for their participation. Participant privacy and confidentiality were protected throughout data collection and analysis, and all data were stored on secure, password-protected systems accessible only to the research team. All figures and screenshots presented in the manuscript depict only the simulation environment and do not contain personal information.

## Results

### Participant Demographics

A total of 12 participants completed the alpha test, and 12 completed the beta test. The demographic composition of each group is summarized in Table 4.

**Table 4:** Demographic and relevant background characteristics of alpha and beta test participants.

Characteristic		Alpha test (n=12), n (%)	Beta test (n=12), n (%)		
Education level					
	Undergraduate	4 (33.3)	10 (83.3)		
	Graduate	8 (66.7)	2 (16.7)		
Prior gaming experience					
	Yes	5 (41.7)	9 (75)		
	No	7 (58.3)	3 (25)		
Prior commercial kitchen work experience					
	Yes	3 (25)	0 (0)		
	No	9 (75)	12 (100)		

### Clarity of Instructions

**Figure 4:**
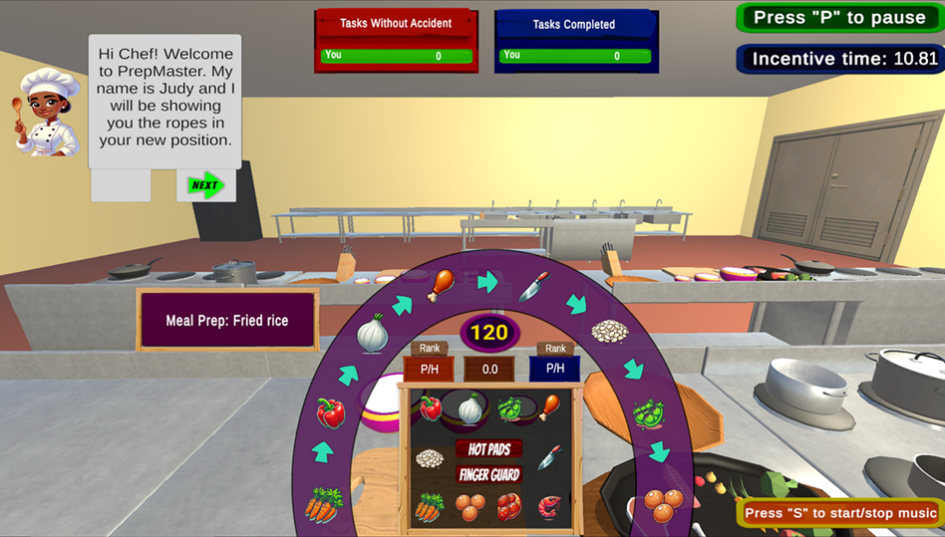
PrepMaster training stage highlighting the virtual assistant Chef Judy, who provides step-by-step guidance on task procedures, safety requirements, and use of the game interface.

In the alpha test version of PrepMaster, participants were introduced to the game through a compulsory training stage. This tutorial involved a static image of the user interface with guided textual instructions delivered by a virtual assistant named Chef Judy. Participants navigated through the instructions without engaging in actual gameplay. The screen was noninteractive, and all game mechanics were explained through written text shown by Chef Judy (see top left corner in Figure 4).

During the alpha test, 5 participants explicitly reported that the instructions were too long, difficult to follow, and hard to remember. They recommended making the instructions more concise and easier to retain. In addition to the interview feedback, researcher observations revealed that participants who did not identify as regular gamers were more likely to struggle with understanding the instructions. These participants often required intervention from one of the researchers to clarify what actions were expected before they could proceed with gameplay.

In contrast, most participants who described themselves as regular gamers reported that the instructions were sufficient. Two of them noted that even if the tutorial was not fully clear initially, the gameplay itself helped clarify expectations. One participant stated, “The instructions were too much for me to remember everything, but I was able to start connecting the dots once I started playing.”

In response to these findings, three major revisions were made.

**Figure 5:**
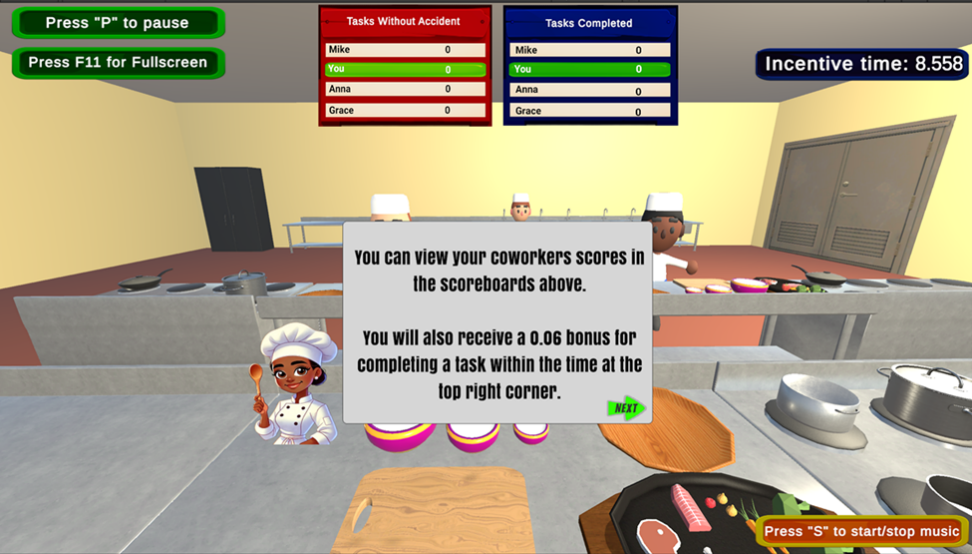
Reminder instructions in PrepMaster informing participants about peer score visibility and the availability of incentives in specific stages.

1. The instructional content was reduced by eliminating redundant details and simplifying the language. Only essential information needed to understand and play the game was retained.
2. The tutorial was redesigned to allow for experiential learning, enabling participants to interact with the game during the training phase. This approach supports stronger learning and memory retention, as experiential methods have been shown to outperform passive instruction in simulation-based environments.
3. Stage-relevant reminders were introduced throughout gameplay to reinforce learning. Before each stage, key instructions were briefly displayed to leverage the recency effect, which can enhance recall and task readiness. For example, Figure 5 shows Chef Judy with a message reminding participants that they can view the scoreboard and the “time to beat” in order to receive the productivity incentive.

Following these revisions, the beta test was used to evaluate the clarity of instructions again. All 12 participants reported that the instructions were easy to understand and that they could remember them after completing the interactive training. A few participants still mentioned that certain aspects became clearer as they progressed, but they noted that the learning curve was manageable and that gameplay reinforced their understanding. These results suggest that the revised training format improved clarity and supported participant comprehension across different experience levels.

### Ecological Validity

#### Overview

The ecological validity of the simulation was evaluated based on the principles of verisimilitude and veridicality, as outlined in prior research [25,27]. These 2 dimensions were examined through the alpha and beta usability tests. In this study, verisimilitude was assessed by asking participants about their perception of how realistically the simulation represented the work environment and tasks. Veridicality was evaluated by examining participants’ perceptions and behavioral responses to the peer effects mechanisms, including both peer presence and peer performance scores. Together, these measures provided a practical means of validating whether the simulation effectively replicated key aspects of real-world social and task dynamics while maintaining theoretical alignment with established definitions of ecological validity.

#### Realism of the Environment and Tasks (Verisimilitude)

Participants in the alpha test generally reported that the visual environment of PrepMaster resembled a real-world commercial kitchen. However, many expressed concerns about the realism of the activities themselves. Several participants described the gameplay as “disconnected,” explaining that it felt more like clicking through static images than performing meaningful kitchen tasks. This distinction highlighted a gap between environmental appearance and the authenticity of player actions.

**Figure 6:**
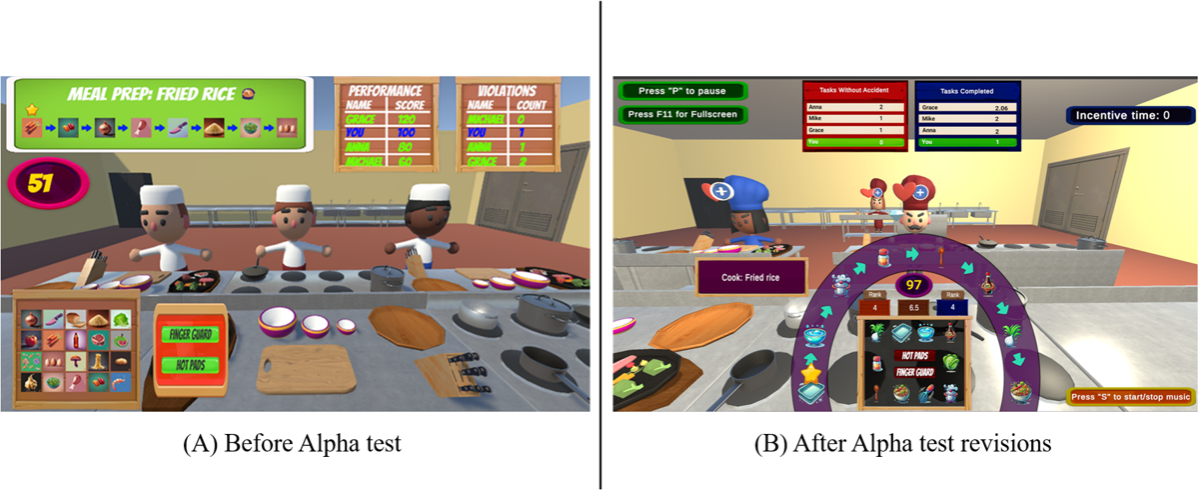
Graphical user interface design (A) before and (B) after the alpha test, showing revisions to the workstation layout, sequence panel placement, and peer score display.

These concerns were especially noted among participants with prior kitchen experience. In the initial graphical user interface (GUI) design (Figure 6A), ingredient images were small and difficult to distinguish, which impeded task performance. One participant noted that the images were too small to interpret quickly. Another explained that the preparation area (ie, the puzzle board) was placed too far from the sequence board displaying meal ingredients, resulting in awkward navigation. This layout made the task feel disjointed and unrealistic. One participant remarked that in an actual kitchen, ingredients are positioned within arm’s reach to support efficient workflow, and the game’s layout did not reflect that logic.

In response to this feedback, the interface was revised to enhance ecological validity. Ingredient images were enlarged, and the positions of the puzzle and sequence boards were adjusted to create a more natural interaction flow (Figure 6B). These changes aimed to make the actions feel more cohesive and reflective of real food preparation tasks.

In the beta test, after these adjustments were implemented, participants reported that both the visual layout and task activities closely resembled those of a real kitchen. Many described the gameplay as intuitive and representative of kitchen activities, with the preparation process now feeling more connected to how they would perform similar tasks outside of the simulation. This suggested that the revised GUI and task design improved the simulation’s ecological validity.

#### Reaction to Peer Effects Design (Veridicality)

Peer effects were induced through multiple visual channels: a real-time scoreboard displaying participant and virtual peer scores, a ranking board showing relative performance, and 4 action icons indicating when a virtual peer completed a task, used safety equipment, incurred an accident, or received an incentive.

In the alpha test, initial feedback revealed visibility issues. A total of 3 of the first 5 participants reported difficulty noticing the scoreboard, ranking board, and action icons during gameplay. As one participant noted, “I noticed that if I moved my head up to look at my score, it would only make me slower, and I would probably not beat them.” Another added, “I was only focused on the meals I had to cook and the timer that showed how long I had. I did not notice anything else.”

**Figure 7:**
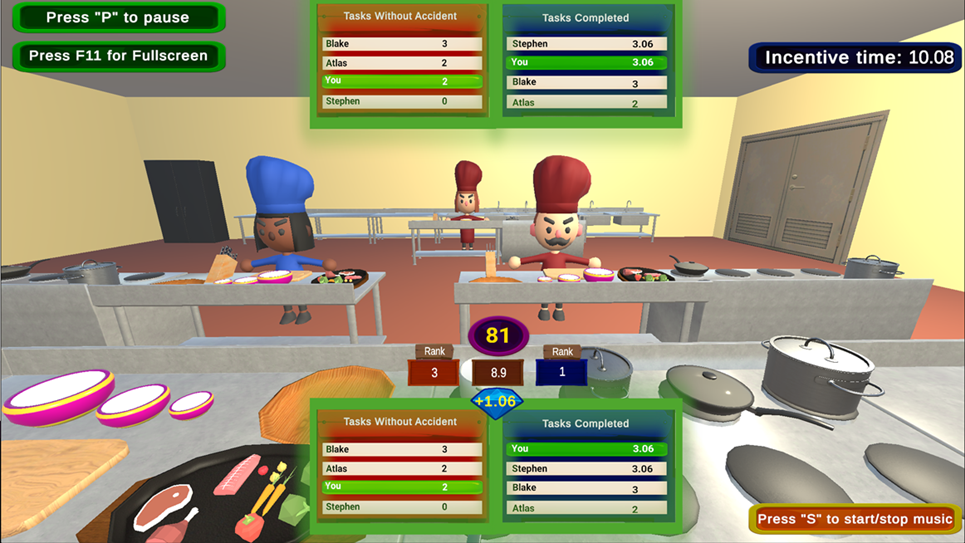
Revised information transmission design implemented after the alpha test, with peer scoreboards displayed at the bottom immediately following task completion.

To address this, the scoreboard was modified to appear at the workstation area for 3 seconds after each task was completed, accompanied by a flashing indicator to capture attention (Figure 7). After this change, all participants, including the 3 who initially reported visibility issues, confirmed they were able to see the scoreboard after each task. However, some still reported missing the ranking board and action icons due to intense focus on task completion under time pressure.

Perceptions of the virtual peers also affected engagement. Of the first 4 participants interviewed during the alpha test, 2 said the virtual characters did not feel real enough, which reduced their motivation to compete. When asked whether they would care about coworker performance in a real kitchen, one said yes, while the other said no. In response, a message was added to the training stage, before each level, explaining that the virtual peers represented real people’s performances. This adjustment increased perceived realism. One participant commented, “I was not sure, but I thought that their scores were based on the previous people that played the game, so I wanted to beat them.”

Feedback also indicated the need to recalibrate the difficulty of peer performance conditions. In the alpha test, 2 participants reported that high peer productivity scores were too difficult and felt unachievable, discouraging them from engaging with the scoreboard. Conversely, low peer productivity scores were seen as too easy, reducing the perceived need to compete. To address this, the scoring algorithms were adjusted so that high and low peer productivity scores were closer to participants’ performance levels, with the aim of making it challenging but still within reach.

A similar issue was observed in the safety performance conditions. Participants exposed to both low and high peer safety information exhibited similar levels of safety behavior. Two potential explanations emerged: (1) the scoring algorithms for high and low peer safety were not sufficiently distinct, or (2) participants used safety equipment so consistently that both high and low peer safety scores (being based on participants’ behavior) appeared similar. To test whether the scoring algorithms for high and low peer safety were not sufficiently distinct, the algorithms were revised to create more polarized safety scores. However, follow-up interviews revealed that participants still did not notice a meaningful difference between the 2 conditions. This supported the explanation that when peer safety scores were tied to participant performance, the system could not generate a sufficiently meaningful contrast.

Before the beta test, this issue was resolved by using predefined peer safety scores that were not influenced by the participant’s behavior. In the beta test, participants clearly observed differences between high and low peer safety conditions. Among the 9 participants who encountered conditions where both peer safety and productivity scores were displayed, 6 reported focusing primarily on the productivity scoreboard. Overall, 2 reported focusing more on the safety scores, and 1 said they focused equally on both.

Additionally, when asked about the extent to which the virtual peers induced peer effects, 9 of the 12 participants reported feeling motivated to outperform the peers, whereas 3 indicated that they did not. Follow-up questioning revealed that 2 of these participants felt that the peer mechanism in the simulation was not sufficiently motivating to elicit competitive behavior, while the third explained that they are generally not competitive, either in games or in real-world situations. Detailed participant responses regarding perceptions of comparison and competitiveness toward the virtual peers are provided in Tables S1 and S2 in Appendix 1.

Together, these findings indicate that when peer performance data are appropriately calibrated and made visible at key moments, nonimmersive virtual peers can elicit competitive responses and social influence. This supports the simulation’s intended peer effect mechanisms as theoretically grounded and behaviorally meaningful.

### Productivity Incentives Design

The performance-based incentive system in PrepMaster awarded a 6 percent productivity bonus when participants completed tasks faster than their personal baseline, which was established during a prior control condition without peer information. Feedback from the alpha test indicated that the incentive was effective in motivating participants to improve their performance.

All 12 participants in the alpha test reported that the bonus was a meaningful motivator. Several stated that seeing the bonus appear on the scoreboard after each task encouraged them to work faster and more efficiently. One participant remarked, “I was even happier when I got a perfect round, that is, when I used safety and still received the bonus.” This suggests that the incentive not only promoted task speed but also reinforced safe behavior, as participants realized they could meet both goals simultaneously.

The beta test confirmed these findings. Participants again reported that the bonus system increased their motivation to perform well. Many described actively monitoring their performance in relation to the incentive threshold and adjusting their strategies to achieve both speed and safety. These consistent results across both tests suggest that the incentive mechanism was well-calibrated and successfully integrated into the overall peer effects and performance design of the simulation.

Overall, results showed that no new usability concerns emerged during the beta test across the evaluated domains of instructional clarity, environmental realism, peer credibility, incentive structure, or information visibility. All previously identified issues from the alpha test were either resolved or substantially mitigated following the implemented revisions. The absence of novel themes and insights in the beta test suggests that saturation had been reached within the scope of the evaluated usability and validity domains. This suggests that the iterative refinements adequately addressed the primary barriers identified during earlier testing.

Following iterative refinements across both testing phases, Table 5 summarizes how the revised system preserved essential environmental fidelity, task demands, instructional clarity, and peer influence mechanisms characteristic of a real commercial kitchen.

**Table 5:** Postiteration alignment of simulation design with real-world commercial kitchen task and peer effects mechanisms.

Elements	Simulation representation	Real-world commercial kitchen context
Environmental fidelity	Nonimmersive 2D environment with simplified prep and cooking layout. Added ambient kitchen-noise audio to approximate environmental context.	Kitchens include prep tables, cooking lines, tools, spatial constraints, and ambient noise, all shaping attention and workflow.
Task demands	Timed (120-second) knife-cutting task emphasizing accuracy and speed.	Fast-paced setting with time pressure, rapid decision-making, and repetitive food-preparation tasks.
Safety behaviors and consequences	Knife sheathing required during noncutting movements. Safety errors can trigger an in-game injury event that introduces a time penalty, delaying task progress and reducing productivity performance.	Knife safety, hazard awareness, and compliance are essential. Unsafe actions can cause injuries, workflow disruption, and social or supervisory repercussions.
Peer effects	Three virtual peers displayed through performance and safety scores to simulate real coworkers. Scores calibrated to realistic ranges; a message in the beta test indicated that peer data came from real participants’ performances, increasing perceived realism and engagement.	Workers adjust effort, speed, and compliance based on coworker behavior. Peer norms shape motivation, competition, and safety climate. Real colleagues provide social relevance and accountability.
Instructional training	Experiential training with interactive tasks and stage-specific reminders.	Workers typically learn through hands-on practice with brief coaching or reminders.

## Discussion

### Overview

This study assessed the usability and ecological validity of a nonimmersive simulation designed to model peer effects on safety and productivity behavior. Across 2 rounds of iterative testing, the findings demonstrated that the simulation was usable and that several mechanisms of peer effects could be meaningfully elicited when key design elements were calibrated appropriately. Participants responded more consistently to peer information when performance scores were realistic, attainable, and clearly presented, and when virtual peers were perceived as credible. Instructional comprehension also improved after shifting from static text to experiential training. Together, these results indicate that a well-designed nonimmersive simulation can approximate components of peer-driven behavioral processes, supporting its use for studying peer effects in controlled environments.

### Integration With Previous Research

The findings highlight several factors that shaped users’ engagement with peer information. Feedback calibration played a critical role in activating competitive responses as participants were more likely to attend to and act on peer performance information once leaderboard visibility was improved and peer scores were adjusted to reflect attainable differences. The findings align with social comparison theory, which suggests that upward comparisons motivate behavioral change when reference standards are perceived as attainable [31,59]. During the alpha test, unrealistically high and implausibly low peer scores reduced engagement. After recalibration to reflect achievable performance differences, participants reported stronger motivation to compete, reinforcing the importance of credible comparison targets when modeling peer effects.

The results also support literature on social presence, which suggests that social influence intensifies when the source of comparison is perceived as a real and intentional other [60,61]. When participants were informed that peer scores were based on prior user performance, perceived realism increased and responsiveness to comparative information strengthened. This demonstrates that subtle cues signaling human origin can meaningfully influence engagement in nonimmersive environments.

Improvements in instructional design further align with research showing that experiential learning enhances comprehension and retention compared to passive instruction [62-64]. The introduction of interactive training and stage-specific reminders likely supported short-term recall processes consistent with memory reinforcement principles [65,66]. These refinements helped ensure that participants understood task requirements before engaging in the virtual environment and also supported findings from prior studies suggesting that, when paired with clear instructions, VR simulations can achieve good usability [67,68].

The structured ecological validity assessment reflects principles of veridicality, emphasizing preservation of task demands, environmental constraints, and action-consequence contingencies that give rise to real-world behavior [25,26]. By maintaining time pressure, safety contingencies, and visible peer performance feedback, this study’s results suggest that the simulation preserved core mechanisms necessary for eliciting peer-driven behavioral responses.

### Study Implications

Methodologically, this study demonstrates that nonimmersive simulations can approximate peer effect processes when grounded in theory and refined through systematic user testing. While immersive VR systems often receive attention for enhancing perceptual realism and presence [69,70], the present findings indicate that behavioral realism does not depend solely on technological immersion. When grounded in strong theoretical foundations and refined through evidence-based feedback and iterative testing, digital media such as nonimmersive virtual systems can also elicit meaningful, realistic behavioral responses [71,72]. By preserving core social comparison mechanisms and task contingencies, this study shows that nonimmersive platforms can offer a low-cost and low-risk method for experimentally examining social processes that are difficult to isolate in real workplaces due to safety constraints and environmental variability.

Practically, this study suggests that organizations and managers may benefit from incorporating calibrated peer information into performance systems as realistic benchmarks, visible comparison metrics, and credible feedback sources may facilitate social comparison and motivation without requiring complex technological infrastructure. The results also indicate that instructional clarity and experiential onboarding are essential for ensuring that performance differences reflect behavioral mechanisms rather than misunderstandings of task expectations.

### Limitations

Several limitations should be considered when interpreting this study’s findings. The simulation modeled unidirectional peer effects, meaning participants could observe virtual peers but could not interact with them. While this was used to mitigate Manski’s [11] reflection problem, it limited the ability to capture more complex social exchanges such as collaboration, verbal persuasion, or feedback loops, which are present in the real world.

Also, all virtual peers in the simulation follow comparable behavioral rules and share similar visual traits. However, in real-world environments, peer effects are shaped by factors such as perceived similarity [73], social identity [74,75], and interpersonal credibility [76]. When virtual peers lack diversity, the simulation may overlook or underrepresent variations in peer effects that arise from differences in individuals’ status, expertise, or interpersonal affinity.

Finally, the sample consisted primarily of university students, which may limit generalizability to other populations [77]. Experienced workers or industry trainees may differ in their familiarity with workplace safety culture, task environments, and gaming interfaces.

### Future Work

An avenue for future research involves how perceptions of virtual peers impact competitive, safety, and task-related behavior by manipulating the content and framing of peer information. Prior studies show that social comparisons depend strongly on how the comparison target is described, including perceived competence, similarity, and expertise [31,78]. In this study, a message indicating that virtual peer scores reflected “realistic performances” increased credibility and engagement. Building on this, future work could vary this message to intentionally elicit upward or downward comparisons. Describing peers as professionals may create an attainable upward anchor that increases effort [54], while labeling them as novices may support downward comparison processes that impact confidence or risk-taking [79]. Systematically manipulating these framing cues could provide insight into how perceived peer competence and similarity shape behavioral responses in virtual simulations.

Another avenue involves examining the competitive dynamics associated with peer information. In this study, some participants noted limited motivation to compete because outperforming virtual peers offered no extrinsic benefit. This matters because performance-based incentives are used in workplaces and training systems, and understanding how they function in simulations is essential for modeling real-world behavior [55]. Prior work shows that incentives increase competitive engagement, especially when performance is evaluated relative to others [80-82]. Future studies could look into whether adding rank-based or performance-contingent incentives enhances competitive motivation in virtual environments and whether these incentives interact with perceived realism to impact safety and productivity outcomes. This could clarify how incentive structures shape peer-driven behavior and help align simulation-based findings with patterns observed in actual work settings.

A more robust experimental design could clarify how virtual peer information shapes both productivity and safety behavior within simulations. Productivity and safety are interdependent; increases in efficiency can elevate safety risk when individuals prioritize speed over safety behaviors [83,84]. Understanding how virtual peers’ performance information impacts this balance is important because peer information can directly shift performance strategies, risk tolerance, and compliance behaviors [4,5,85]. A larger sample would allow researchers to identify mechanism-level effects and test how different patterns of peer performance information drive these behavioral outcomes, aligning with calls for more powered studies in simulation-based peer effects research [16] and VR-based behavioral modeling [25].

### Conclusion

This study demonstrated that nonimmersive virtual simulations can be used to model peer effects on safety and productivity behavior when designed with theoretically grounded, user-centered features. These findings are consistent with prior research demonstrating that virtual simulations and serious games can achieve ecological validity and usability when grounded in theory and empirically evaluated [71,86-88]. Beyond supporting existing literature, the study expands the methodological tools available for examining peer-driven behavior in controlled settings where real-world experimentation may be impractical or unsafe. The findings also suggest that organizations designing performance feedback systems may benefit from grounding peer comparison structures in established social and behavioral theory. Specifically, this study’s findings suggest that realistic and attainable benchmarks, clearly presented comparative metrics, and credible feedback sources may enhance motivation and behavioral responsiveness without requiring complex technological infrastructure.

## Appendix

Multimedia Appendix 1 Participant responses to peer competitiveness question and select participant responses to real-world competitiveness question.

## Abbreviations

GUI: graphical user interface
IRB: institutional review board
VR: virtual reality

## Data Availability

The datasets generated and analyzed during this study are available from the corresponding author upon reasonable request.
