# Supplementary material for: Peer Effects on Safety and Productivity in a Simulated Workplace Through Usability and Ecological Validity Testing of a Nonimmersive Virtual Simulation With University Students: Cross-Sectional Qualitative Study
Source: JMIR Form Res. 2026 Jul 31;10:e81061. doi: 10.2196/81061 (PMC13476730; doi:10.2196/81061)
Supplement: Supplementary file 1 [file formative_v10i1e81061_app1.docx]

**Table S1:** Participant responses to peer competitiveness question

| Question: Did you feel motivated to adjust your performance after viewing the virtual peers’ performance? | | |
| --- | --- | --- |
| Participant | Verbatim Response | Outcome |
| P01 | “It did kind of add some competitiveness to it.” / “I was trying to see how they were doing versus how I was doing.” / “Yes, I wanted to beat them.” | Competitive |
| P02 | “When I noticed I was actually doing good, I was like, oh cool, I’m gonna try to do better.” / “Sometimes I was ahead of them… might as well go for first.” | Competitive |
| P03 | “I just focus on my score.” / “I just wanted to get the bonus.” | Not competitive |
| P04 | “I knew they were there but never looked after them.” / “It was more boring; maybe I’d care more if it showed whether you win or lose.” | Not competitive |
| P05 | “It wasn’t easy… but this encourages me to finish more instead of finish fast.” / “The ranking reminded me to do better.” | Competitive |
| P06 | “When I saw it, I wanted to do better.” / “Wanting to catch up distracted me.” / “It was the bonus more than the co-workers.” | Competitive |
| P07 | “When compared to others, I worked harder.” / “I wanted to beat my co-workers more.” / “Without co-workers, I tried the least.” | Competitive |
| P08 | “Eventually it felt very competitive.” / “I was trying to pass them.” | Competitive |
| P09 | “I wanted a perfect score without accidents, more of a personal competition.” / “I was not really bothered about the leaderboard.” | Not competitive |
| P10 | “When they were around, I tried to get the highest score.” / “Yes, especially when I saw co-workers collecting bonus—it motivated me.” | Competitive |
| P11 | “It was a good gauge, if someone did it faster, I should aim for that time.” / “It wasn’t about being first, just benchmarking.” | Competitive |
| P12 | “I definitely wanted to beat them.” / “Working with co-workers made it more motivating.” / “When there weren’t any co-workers, it was boring.” | Competitive |

**Table S2:** Select Participant responses to real-world competitiveness question

| Question: Given a similar scenario in the real world, would you feel motivated to adjust your performance? | | |
| --- | --- | --- |
| Participant | Verbatim Response | Interpretation |
| P03 | “Not really. I just kind of focus on doing my thing, not so much trying to beat people.” | Low social competitiveness; focuses on self-performance rather than comparison. |
| P04 | “No, I wouldn’t say I’m competitive. Unless there’s something to win, I just do my part.” | Not competitive unless extrinsic rewards are present. |
| P09 | “If maybe I’m outperforming them… no rewards too. If there’s no reward, why am I competing?” | Not competitive unless extrinsic rewards are present. |
